# Supplementary figures and images for: Place, displacement, and health-seeking behaviour among the Ugandan Batwa: A qualitative study
Source: PLOS Glob Public Health. 2024 Jun 12;4(6):e0003321. doi: 10.1371/journal.pgph.0003321 (PMC11168611; doi:10.1371/journal.pgph.0003321)

**S1 File. Health-seeking behaviour excerpt from semi-structured interview guide.**


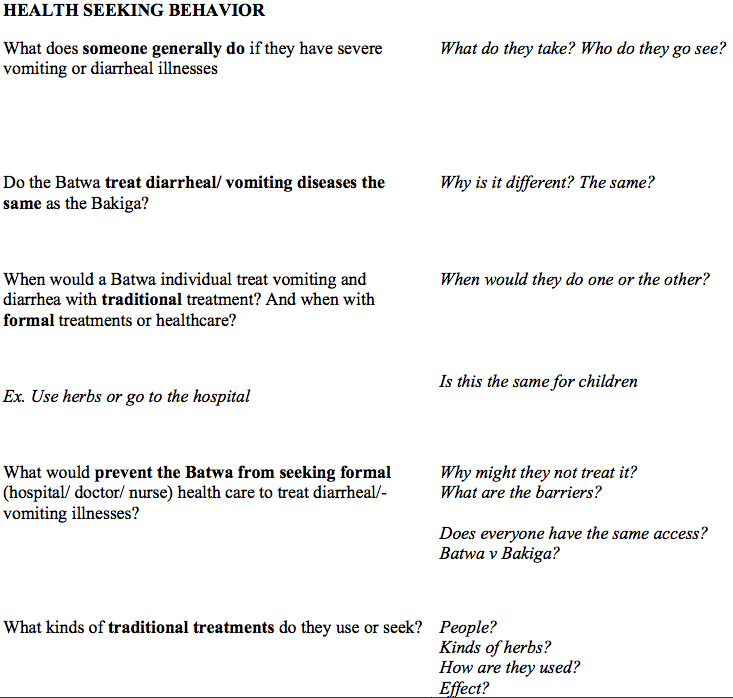

Supplement: S1 File — (DOCX) [file pgph.0003321.s002.docx]

**S2 File. Health-seeking behaviour excerpt from focus group interview guide.**

***
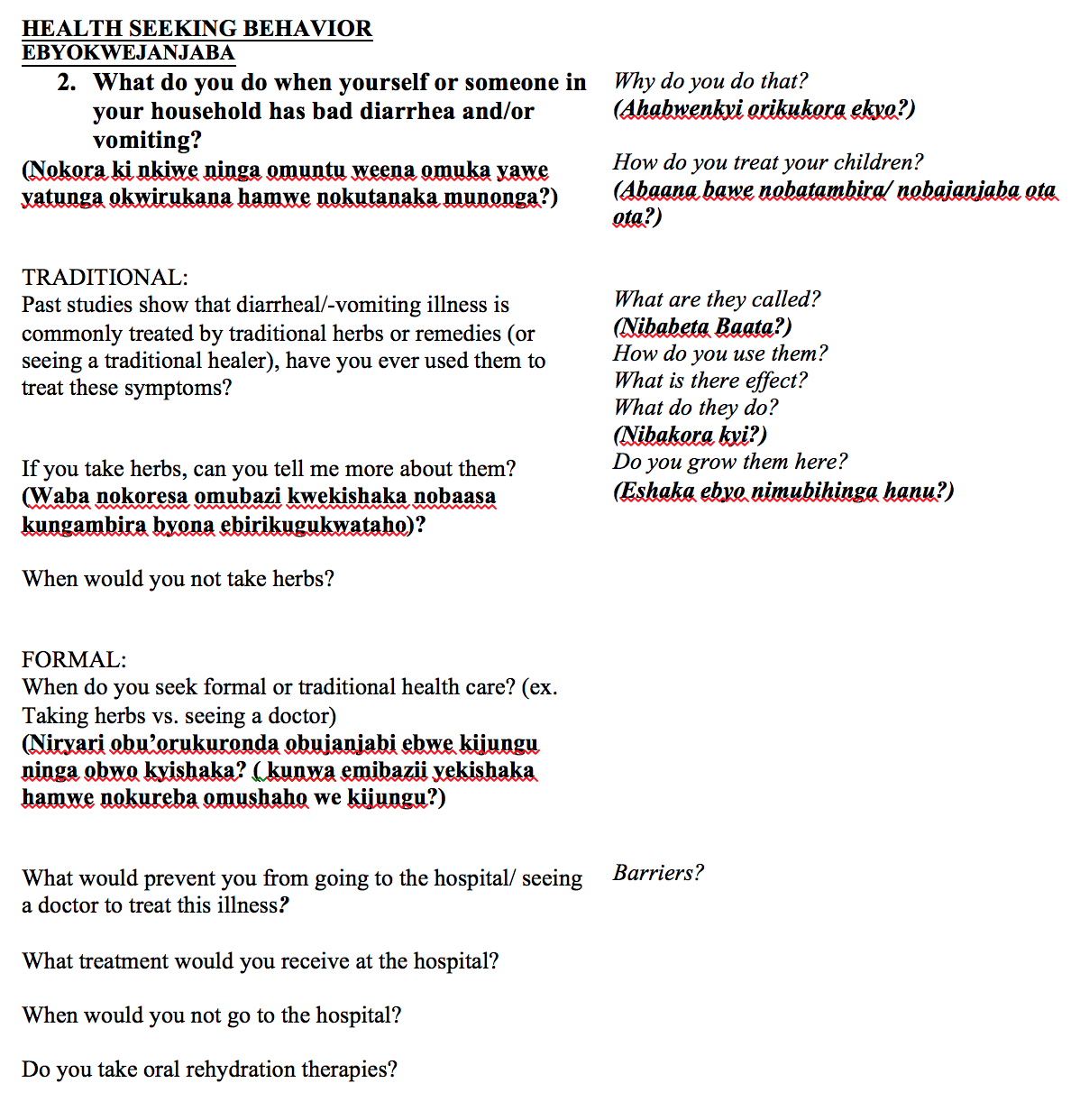
***

Supplement: S2 File — (DOCX) [file pgph.0003321.s003.docx]
