# Supplementary material for: Place, displacement, and health-seeking behaviour among the Ugandan Batwa: A qualitative study
Source: PLOS Glob Public Health. 2024 Jun 12;4(6):e0003321. doi: 10.1371/journal.pgph.0003321 (PMC11168611; doi:10.1371/journal.pgph.0003321)
Supplement: S3 File — (DOCX) [file pgph.0003321.s004.docx]

**S3 File. Additional, salient quotations on health-seeking behaviour for acute gastrointestinal illness in the context of Batwa connection to place and displacement.**

| Batwa Health-Seeking Behaviour in Response to Acute Gastrointestinal Illness: Additional Exemplar Supporting Interview Quotations | |
| --- | --- |
| Theme | *Quotations* |
| Transition from “Place” and Reflections on Health in the Forest | *• “In the forest we would have food items like meat that we can’t get now. Secondly we are not allowed to go back to the forest. We don’t have time to do that yet we would want to. Because we have special things that we would get from the forest like ebihama and ebikwa. We can’t have such foods in the village and other herbs. Another thing that we would have while we were in the forest is honey and nyakibazi and rukokota for treating snakes of the stomach [worms]. It’s hard to get such things today but if we would get permission we would go back to the forest and find them.“*  *• “When we would fall sick we would just move around the forest and look for the herbs that we would take and get better.”*    *• “We are regretting that we were moved out of the forest because we can’t get the same kind of food anymore. In the forest, we used to have a lot of food that was enough to feed everybody and we would never fall sick. Whenever we would take a bitter herb to treat any illnesses, we would get cured there and then.”*  *• “For us here we are all young. But for the little things I heard from my parents and grandparents the time they were staying in the forest death was not common. But when we moved out of the forest we started eating different kinds of food that were brought about by whites. And we started suffering from certain diseases. We stopped eating the kinds of foods we would eat while we were in the forest and foods that were medicine. That’s why we don’t have better health. But if we would get back to using foods and fruits or medicine in the forest and ebihama or honey we would have a mutwa health of children not falling sick and having better health. But when we stopped eating all that and with the change of the government batwa are really dying. Me I see that there is a great change since we moved out of the forest. Because we had food for medicine.”*  *• “The situation is hard now the situation was better in the forest because we used to have good food. Meat was there and mud fish all the time for malaria whenever we would get sick Mukula (herb) was there to treat it. Roasted meat and I would have my honey. There were even wild pigs. We would just find any kind of food. But now we first suffer under sun to get food. We really used to eat good food. If you are eating ebihama, ebikwa you can’t have bad health. Recently I found ebihama and ate them but they were not good to me because they made me have diarrhea. I see that as a problem and think the situation is good now. These days I eat good food from the village and I can’t find that good food from the forest.”*  *• “In the past when we were in the forest we never had enough food because we would only eat what we would land on. We used to have a lot of food that we miss today like ebihama, honey, and other herbs. The food that we would eat while in the forest was nutritious. Whenever we would eat it we would not get hungry very fast like today. In fact you would have a meal and wait for like 2days before eating again and you would not even feel hungry.”*  *• “We used to have enough food but now we are suffering with digging. If it were those days I would now be moving around and picking my kikwa(yams) for food. We would never bother looking for drugs because we had nyakibazi (local herbal medicine) that would take and not get sick. In the past batwa would never die.”*  *• “To add on this a mutwa used to stay in the forest and we had all the peace that we wanted. We were respected and had better health compared to today now that we are out of the forest. In the forest we used to eat different kinds of food like ebikwa, ebihama, honey and meat. Meat that was got by hunting and from a wild animal would be meat for medicine (it had medicinal qualities). All that would keep us strong and healthy.”*  *• “But if we were to get land and ask from the park some plants that we used to eat we could come and grow them in our own land we could start feeding on them and there we would have better health than we have now.”*  *• “In the forest we used to eat ebihama. The situation was too good. I even wish to go back because there are good things. There are foods that strengthen the body. Even local herbs are there.”* |
| ‘Intellectual Access’ to Care | *• "It's hard to get some herbs now (ebihama) so that you can teach them to our children on how to even plant them or even get them from other places."  • "The new generation will not know where to get the herbs from...most of their children do not know about the herbs and they believe that in the near future none will be knowing about them or how to use them."*  *• "Because they have mixed up local herbs with modern medication- we actually don't know when we will settle in terms of health."* |
| Batwa Identity and Way of Life | *• "Our parents and grandparents used to take herbs whenever they are sick, so we have to take up the tradition- even before hospitals were set up we could take herbs to treat certain illnesses and we would actually get well."  • "We actually want our children to know about the herbs so that in future when we are not still surviving they can treat some diseases on their own."*  *• "Sometimes vomiting and diarrhea are due to witchcraft and herbs don't treat the witchcraft- sometimes you can take such herbs and die." (socio-cultural and cosmological beliefs)* |
