# Supplementary material for: Place, displacement, and health-seeking behaviour among the Ugandan Batwa: A qualitative study
Source: PLOS Glob Public Health. 2024 Jun 12;4(6):e0003321. doi: 10.1371/journal.pgph.0003321 (PMC11168611; doi:10.1371/journal.pgph.0003321)
Supplement: Attachment — Submitted filename: Response to Reviewers.docx [file pgph.0003321.s006.docx]

**Manuscript ID: PGPH-D-23-02190:** *“Place, displacement, and health-seeking behaviour among the Ugandan Batwa: A qualitative study”*

**Editor Comments:**

**Thank you for submitting this article addressing an important public health issue among Indigenous Peoples in rural Uganda. Forced displacement and land dispossession can impact people’s lives in several ways. This study particularly explored Batwa’s connection to place and their health-seeking behavior for acute gastrointestinal illness using qualitative methods. In addition to reviewer’s comments, here are some questions and comments that would help improving the structure and discussion of this manuscript:**

Author’s Response: Thank you for your insightful questions and suggestions. We have responded point-by-point to each of your comments below.

**Two Research Ethics Boards (at McGill University and University of Guelph) based in Canada reviewed this article. Why not IRB from a University based in Uganda?**

Author’s Response: Thank you for this question. At the time of this study, the Ugandan IRB (via the Ugandan National Council of Science of Technology) was not functioning and ethical approvals were not available. We also consulted with the Director for Graduate Research at Makerere University (Uganda's biggest and top university) at the time of the study, who indicated that there were no ethics approval board or guidelines at Makerere University. We were advised to proceed with the ethics approvals from other countries.

**How local universities and/or researchers participated in the conduction of this research. Is any coauthor from or located in Uganda?**

Author’s Response: Thank you for this important question. Yes, three co-authors are from or located in Uganda (as reflected in their affiliations) and four local, community researchers were also involved in facilitation and interpretation during data collection (as discussed in the Acknowledgements). Additionally, on page 6 (Methods section, “Partner communities and research approach”), we have indicated that the research involved collaboration among *“individuals representative of Batwa communities, governmental and non-governmental organizations, as well as academic researchers from Uganda, Canada, and the UK”.* The Uganda-located organizations and academic institutions are also depicted in Figure 1 (i.e., Makerere University, Uganda National Health Research Organization, Ugandan Ministry of Health, Bwindi Community Hospital, Batwa Development Program, and the 10 Batwa communities who partnered in this research).

**I suggest checking for repetition in results and discussion sections. Also, the focus in ‘gastrointestinal illnesses’ seems to be secondary in a large proportion of the results/discussion.**

Author’s Response: Thank you for this suggestion. We have made minor revisions to avoid redundancy between these sections. Thank you for your insightful comment related to the ‘focus’ on acute gastrointestinal illness (AGI) being secondary in the results/discussion. Indeed, we intended for AGI to be a ‘lens’ or ‘focal point’ through which to explore the dynamics between place, displacement, and health-seeking behaviour for Batwa. As you pointed out, AGI was of secondary interest to us, in that it provided an analytic frame or anchor point from which to explore our primary interest: interconnections between the broader concepts of place, displacement, and health-seeking behaviour.

To clarify this in the text, we have made a change in the introduction (pg.4, line 109; new text is underlined and italicized):*“In order to explore the dynamics between place, displacement, and health-seeking behaviour for Batwa, this study ~~focused on~~ examined experiences with acute gastrointestinal illness, involving diarrhea and/or vomiting [28]”*

**Lack of access to water, sanitation and hygiene services are very important risk factors for gastrointestinal illnesses. Did the authors explore access to these services or other environmental risk factors?**

Author’s Response: Thank you for this question. Exploring access to these services and other environmental risk factors was beyond the scope of the present study’s objectives; however, these important factors were examined in four companion articles published as part of the broader project this present study was connected to. We have cited these articles in the introduction and discussion (references #: 27, 30, 31, 41 in the manuscript, and listed below)

- Brubacher LJ, Berrang-Ford L, Clark S, Patterson K, Lwasa S, Namanya DB, et al. ‘We don’t use the same ways to treat the illness:’ a qualitative study of heterogeneity in health-seeking behaviour for acute gastrointestinal illness among the Ugandan Batwa. Glob Public Health. 2022;17(8):1757–72.
  - A qualitative study focused on different aspects of health-seeking behaviour (i.e., motivations for seeking care), and not in relation to place or displacement of Batwa from ancestral forest lands.
- Clark S, Berrang-Ford L, Lwasa S, Namanya DB, Edge VL, IHACC Research Team, et al. The burden and determinants of self-reported acute gastrointestinal illness in an Indigenous Batwa Pygmy population in southwestern Uganda. Epidemiol Infect. 2015;143(11):2287–98.
  - A quantitative study focused on the burden of self-reported AGI and association with socio-demographic and environmental variables.
- Patterson K, Clark S, Berrang-Ford L, Lwasa S, Namanya D, Twebaze F, et al. Acute gastrointestinal illness in an African Indigenous population: the lived experience of Uganda’s Batwa. Rural Remote Health. 2020;20(1).
  - A mixed-methods study focused on perception of illness severity and AGI risks and preventative behaviours.
- Busch J, Berrang-Ford L, Clark S, Patterson K, Windfeld E, Donnelly B, et al. Is the effect of precipitation on acute gastrointestinal illness in southwestern Uganda different between Indigenous and non-Indigenous communities? PLoS One. 2019;14(5):e0214116.
  - A quantitative study focused on specific meteorological variables and association with AGI among Indigenous Batwa and non-Indigenous Bakiga communities.

**As I understand from this study, Batwa’s People used to practice their own medicine until were forced to leave their land. However, in the discussion section of “Batwa identity and way of life, and ‘intellectual access’ to care”, ‘Indigenous forms of healthcare’ were described as ‘complements’ to biomedical systems and not as their own system. I suggest being careful assuming the hierargical relationship between both healthcare systems.**

Author’s Response: Thank you for flagging this concern. We have rephrased this sentence in the discussion (p.15, lines 377-382) to make it more clear that we are suggesting recognition of Indigenous healthcare systems in their own right and alongside (i.e., in concert with) biomedical systems, not as inferior to. The new sentence reads as follows, with new text *italicized and underlined*:

*“These results on Batwa identity and Indigenous treatment knowledge suggest that additional efforts may be required to support Batwa in locating Indigenous herbs outside of the forest, to recognize Indigenous ~~forms of healthcare~~ healthcare systems as important ~~complements to~~ in their own right and alongside biomedical systems, and to consider further integration of Indigenous and biomedical approaches to treatment in the district [27,63].”*

**International recommendations about environmental health and animal protection argue that Indigenous community’s participation in environmental conservation is key to ensure sustainability. About 80% of all biodiversity in the world is protected by Indigenous Communities. The conservative initiative to ensure protection of a declining mountain gorilla population is a key element for this study and the Batwa’s Community and requires more attention in the manuscript. I suggest incorporating more details in introduction and discussion about the role the World Bank/Global Environment Facility Trust Fund is playing in this process and the decision or the possibility to let this population come back to their land. Particularly, in the discussion section, the authors could incorporate structural determinants of health involved.**

Author’s Response: Thank you for this suggestion. In the introduction, we have cited a recent 2023 article published in PLOS Global Public Health by Kokunda and colleagues, which outlines in much more detail the role and shortcomings of the World Bank/Global Environment Facility Trust Fund in relation to Batwa communities. This article has substantially more focus on conservation policy and its impact on Indigenous Peoples globally, and Batwa specifically. This goes beyond what we aim to – and are sufficiently able to – address in our manuscript, which focuses on the connections between Batwa place/displacement and health-seeking behaviour.

We have made revisions to the discussion as follows:

- “Access to the Forest” section (pgs.15-16, lines 395-400): We have integrated additional reference to the Kokunda et al. (2023) article in relation to a recommendation for increasing access to the forest for Batwa: *“As stated by the WHO commission on social determinants of health, this may require “[restoration] of land necessary for sustaining Indigenous culture and livelihoods” [66:2] or Batwa Landback and returning to the forest, as discussed by Kokunda and colleagues [19].”*
- We discussed structural determinants of health in the “Continued advancement of community-based and community-led organizations” section of the discussion (pg.16), though had not explicitly named these determinants as such. Accordingly, we have revised pg.16 lines 403-405 to use the language of structural determinants of health explicitly: *“To develop contextually-appropriate and effective policy or programmatic interventions, it is necessary to understand the scale, from local to global, at which “contextual processes” – structural determinants of health – operate, and at what scale their impacts are felt [7:1833]”.*

**I wonder if letting the Batwa’s People come back to their ancestral land is a recommendation that can potentially impact their health-seeking behavior for acute gastrointestinal illness. I suggest discussing this point.**

Author’s Response: Thank you for this suggestion. We have revised the “access to the forest” paragraph of the discussion (pg.15, lines 393-395) to more explicitly state that access to ancestral lands might shape Batwa health-seeking behaviour for acute gastrointestinal illness:

*“Acknowledgement and respect for Batwa land rights, and, fundamentally, the right to self-determination, could have a significant impact on Batwa health and health-seeking behaviour for acute gastrointestinal illness and more broadly [64,65].”*

Thank you for your insightful and constructive feedback on our manuscript.

**Reviewer #1: The manuscript brings forth the challenges faced by the Indigenous peoples, especially in the present context where numerous transitions are taking place globally including the areas predominantly inhabited by Indigenous peoples. These transitions are affecting the Indigenous communities, making them more marginalized from the mainstream society. Therefore, it is important to address the challenges faced by them for effective formulation of context-specific policy as pointed out by the authors of this manuscript. However, there are a few comments below that the authors need to address as deemed appropriate.**

Author’s Response: Thank you for your helpful feedback on our manuscript. We have responded point-by-point to each of your comments below.

**Abstract and Introduction:
1. The authors have missed sharing the information on the incidence rate or burden of acute gastrointestinal illness among the Batwa communities. Why is it a critical public health issue among the Batwa communities?**

Author’s Response: Thank you for this helpful comment. We have added the following text to the introduction (pg.5, lines 115-118) in order to describe the burden of acute gastrointestinal illness among Batwa:

*“For Batwa, acute gastrointestinal illness has been self-identified as a key health priority [22]. Recent studies have reported a high burden of acute gastrointestinal illness among Batwa [30], particularly in children under three years of age (14-day prevalence = 11.3%), but also diverse health-seeking behaviour in response [27].”*

**Methods:
2. Partner communities and research approach: Kanungu district includes 47 private or government-funded health facilities that range in the level of care they offer- This sentence is not clear.**

Author’s Response: Thank you for this comment. We have clarified this sentence as follows (pgs.5-6, lines 134-139, new text is *italicized and underlined*):

*“Kanungu district includes a total of 47 health facilities (including private and government funded) that ~~range~~ vary in the level of care they offer, from small healthcare centres with primary healthcare capacities to two hospitals that provide specialized treatments and surgeries”*

**3. Data collection and analysis: Please describe the socio-economic background of participants-especially those from the communities.**

Author’s Response: Thank you for this suggestion. For this current study, we did not collect data pertaining to the socio-economic background of participants; however, sociodemographic data was gathered among households in the same 10 communities/settlements as part of the larger project this study is associated with. As such, we have added information about the socio-economic background of participants, based on data from the larger project:

Data collection and analysis section (pg.7, lines 164-166): *“Sociodemographic data collected as part of the larger study [30] indicated low socioeconomic variation among households from the ten settlements and typically no education or primary-level education among households.”*

**Results:
4. It is also important to add the perceptions of the Batwa communities on acute gastrointestinal illness- for instance, what is known locally in the community, their understanding of the illness and when they decide to seek care- their perceptions about the severity of the diseases- if the information on the same has been collected by the researchers.**

Author’s Response: Thank you for this insightful comment. As part of the present study, we did not gather this information. However, data pertaining to Batwa perceptions of the severity of acute gastrointestinal illness and more findings related to decisions around when and how to seek care are reported in two companion papers that we have cited in the introduction and discussion: References #31 and 27, respectively (also listed below):

- Patterson K, Clark S, Berrang-Ford L, Lwasa S, Namanya D, Twebaze F, et al. Acute gastrointestinal illness in an African Indigenous population: the lived experience of Uganda’s Batwa. Rural Remote Health. 2020;20(1).
- Brubacher LJ, Berrang-Ford L, Clark S, Patterson K, Lwasa S, Namanya DB, et al. ‘We don’t use the same ways to treat the illness:’ a qualitative study of heterogeneity in health-seeking behaviour for acute gastrointestinal illness among the Ugandan Batwa. Glob Public Health. 2022;17(8):1757–72.

**5. It is important to have uniformity while writing the quotes of each participant. Participant age, gender, and educational status may be mentioned after the quotes.**

Author’s Response: Thank you for this comment. Throughout the results, we have revised the text to ensure uniformity while writing quotes. That is, we have consistently indicated the gender of the participant at the end of each quote, rather than in the lead-up to the quote.

We did not gather data on a given participant’s age or educational status: We only have sociodemographic data from these 10 settlements from the larger project we have referenced (which this current study was part of), but it is not linked to the qualitative data in this study. As such, we are unable to include that information after each quote. The reason we have data on gender for each quote is because data were gathered in separate male and female focus group discussions.

Thank you again to the editor and reviewer for their thoughtful and constructive review of our manuscript. We believe the changes we have made in response have strengthened this manuscript.
